# Comparison of Kidney Graft Function and Survival in an Emulated Trial With Living Donors and Brain-Dead Donors

**DOI:** 10.3389/ti.2024.13208

**Published:** 2024-08-29

**Authors:** Emilie Savoye, Gaëlle Santin, Camille Legeai, François Kerbaul, François Gaillard, Myriam Pastural

**Affiliations:** ^1^ Direction Prélèvement Greffe Organes-Tissus, Agence de la Biomédecine, Saint-Denis La Plaine, France; ^2^ Service de Transplantation, Néphrologie et Immunologie Clinique, Hôpital Edouard Herriot, Hospices Civils de Lyon, Lyon, France

**Keywords:** living donation, kidney function posttransplant, emulation target trial, age, brain-dead donor

## Abstract

Living donation (LD) transplantation is the preferred treatment for kidney failure as compared to donation after brain death (DBD), but age may play a role. We compared the 1-year estimated glomerular filtration rate (eGFR) after kidney transplantation for recipients of LD and DBD stratified by recipient and donor age between 2015 and 2018 in a matched cohort. The strength of the association between donation type and 1-year eGFR differed by recipient age (*P*_interaction_ < 0.0001). For LD recipients aged 40–54 years versus same-aged DBD recipients, the adjusted odds ratio (aOR) for eGFR ≥60 mL/min/1.73 m^2^ was 1.48 (95% CI: 1.16–1.90). For DBD recipients aged ≥ 60 years, the aOR was 0.18 (95% CI: 0.12–0.29) versus DBD recipients aged 40–54 years but was 0.91 (95% CI: 0.67–1.24) versus LD recipients aged ≥60 years. In the matched cohort, 4-year graft and patient survival differed by donor age and type. As compared with DBD grafts, LD grafts increased the proportion of recipients with 1-year eGFR ≥60 mL/min/1.73 m^2^. Recipients aged ≥60 years benefited most from LD transplantation, even if the donor was aged ≥60 years. For younger recipients, large age differences between donor and recipient could also be addressed with a paired exchange program.

## Graphical Abstract

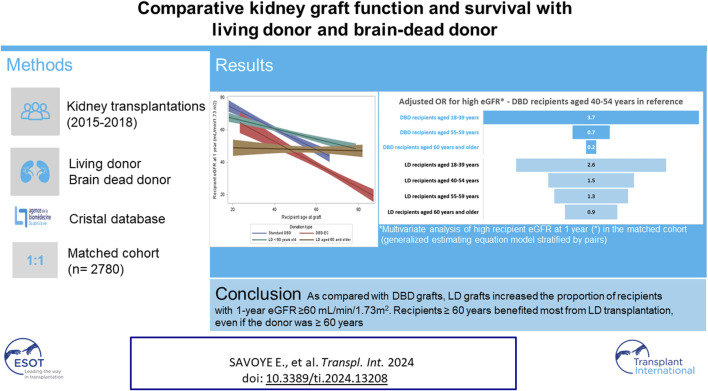

## Introduction

Graft and patient survival with living donation (LD) is better than donation after brain death (DBD) [1–6]. Data from a UK transplant registry showed that all-cause mortality was lower for recipients of older LD kidneys (aged ≥60 years) than standard-criteria DBD (DBD-SC), but graft and overall survival were lower for LD recipients with older living donors rather than younger (aged < 60 years) [7]. DBD and LD transplantations have significant differences that may affect post-transplantation survival. One of the major advantages of LD transplantation is that it allows for pre-emptive transplantation. The age and immunological profile of LD recipients may also differ from those of DBD recipients.

At first glance, a short cold ischemia time and very good health of the donor seem to result in higher eGFR after LD than DBD transplantation. Alternatively, these expected benefits of LD over DBD may be counterbalanced by the better age and immunological matching between the donor and recipient in DBD than in genetically and emotionally related LD. Altogether, LD and DBD have different graft access procedures and clinical characteristics that may affect the outcomes of kidney transplantation (KT) independent of the donation type [1].

From our annual medical and scientific report [8], for 44% of LD recipients, the eGFR at 1 year was >60 mL/min as compared with 51% for DBD-SC recipients. This unfavorable outcome for LD prompted us to conduct this study.

In the context of the various pros and cons for each of the two strategies, we compared the impact of DBD and LD on eGFR at 1 year after transplantation using propensity score (PS) matching to attempt to mimic a randomized trial [9]. Because age is an important element in the choice of donor and eGFR interpretation, we conducted several sensitivity analyses to explore this confounding factor. We analyzed eGFR with DBD and LD by recipient age and donor subgroup, namely, standard criteria and expanded criteria. Secondary outcomes were graft and patient survival at 4 years.

## Materials and Methods

### Patients

We included all LD and DBD first single-organ kidney transplants performed in metropolitan France from 2015 to 2018. We excluded transplants with human leukocyte antigen (HLA) or ABO incompatibility, pediatric recipients, recipients who died in the first week after the transplant, and those with missing data for 1-year eGFR.

### Outcome Measures

**FIGURE 1:**
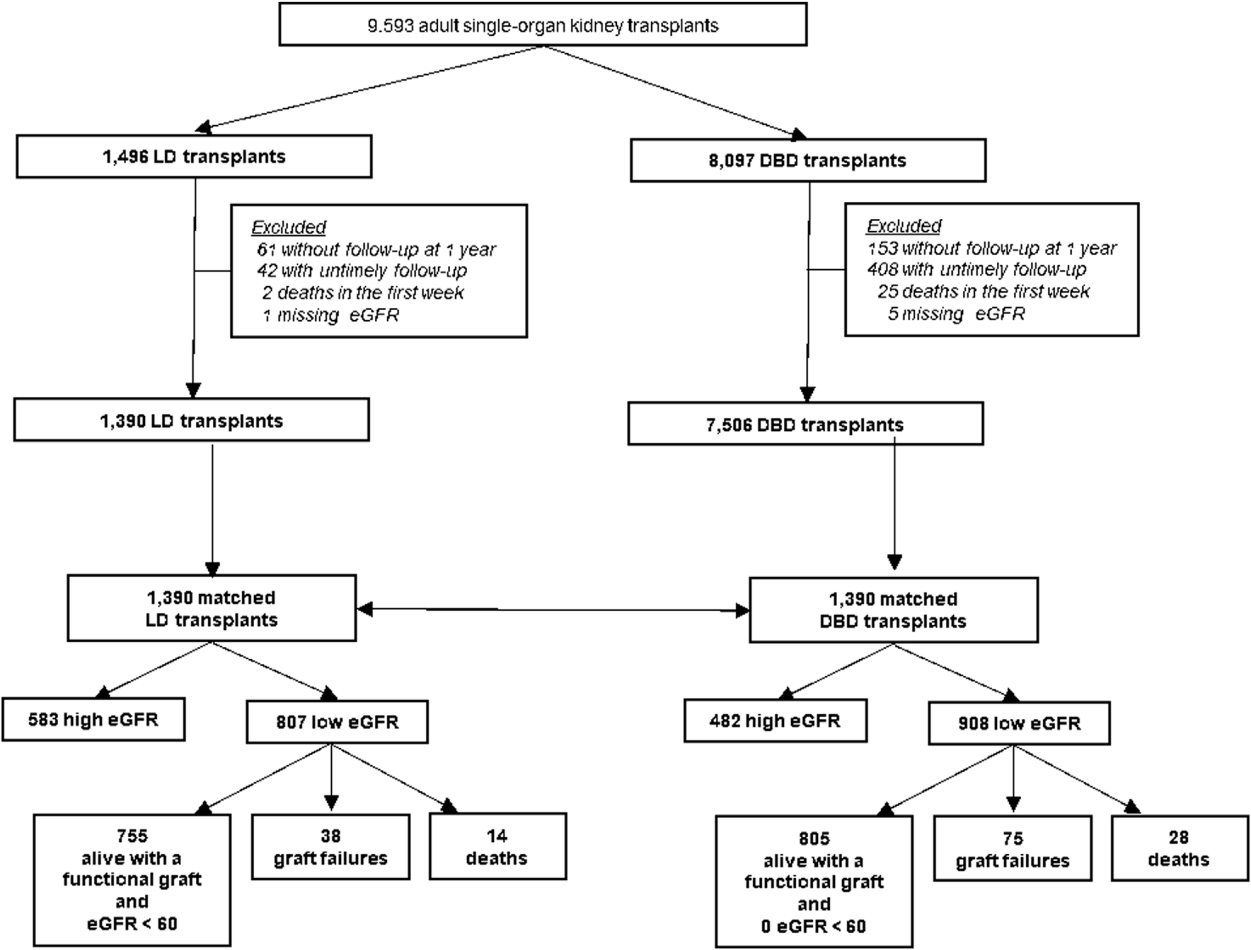
Enrollment and outcomes. eGFR: glomerular filtration rate estimated with the CKD-EPI formula mL/min/1.73 m^2^, LD, living donation; DBD, donation after brain death.

The primary outcome was an eGFR estimated with the chronic kidney disease (CKD)-EPI equation [10] of ≥60 mL/min/1.73 m^2^ or over at 1 year after KT, which corresponds to a normal or mild loss of kidney function according to the international classification of CKD stages. Recipients with graft failure <1 year after KT (n = 113) were classified in the group with eGFR <60 mL/min/1.73 m^2^, as were those who died <1 year after KT (n = 42), because death is most often pooled with graft failure in graft failure analysis. When eGFR was measured <9 months or >21 months after the KT or was > 150 mL/min/1.73 m^2^, 1-year eGFR was considered missing (data missing for 8% of LD and 7% of DBD transplants, detailed in Figure 1). Two additional 1-year eGFR thresholds were explored: 45 mL/min/1.73 m^2^, which corresponds to normal or mild to moderate loss of kidney function (CKD stage 1 to 3a), and 80 mL/min/1.73 m^2^, which we considered as normal kidney function since too few patients in the current study had a 1-year eGFR of ≥ 90 mL/min/1.73 m^2^ (CKD stage 1).

Secondary outcomes were 4-year graft and patient survival.

### Study Variables

Our main studied variables were type of donation (DBD or LD) and recipient age.

We also considered other recipient characteristics at KT: blood group, sex, cardiovascular comorbidities, end-stage renal disease (ESRD) cause, duration of dialysis before KT, BMI, and immunization level assessed by calculated panel-reactive antibodies (cPRA) (0%, 1%–84%, 85%–100%).

Four of these variables were continuous and were categorized. Age cut-offs were chosen according to French kidney allocation rules. BMI, duration of dialysis, and immunization rate cut-offs were chosen according to clinical relevance and statistical criteria (association between outcomes and continuous variables analyzed graphically with restricted cubic splines).

### Data Collection

Data were retrieved from the French national transplant registry, CRISTAL. The French biomedicine agency (*Agence de la Biomédecine*) is a public institution supported by the French ministry of health. One of its missions is to manage organ and tissue procurement and transplantation in France. For this purpose, the CRISTAL registry prospectively collects demographic, clinical, and laboratory data for all organ transplant recipients and donors as well as transplant outcomes in France. The CRISTAL registry has full coverage of all French transplant units. Data are recorded at registration (placement on a waitlist), procurement, and transplantation and annually thereafter. Data collection is mandatory, and research technicians double-check its completeness and accuracy. In accordance with French law, research studies based on this national registry are part of transplant assessment and do not require additional institutional review board approval. The database has been reported to the French National Commission on Computing and Liberty.

### Statistical Analysis

Characteristics of recipients and donors are described with mean (SD), median (inter-quartile ranges) for continuous variables, or number (percentage) for categorical variables. Missing data (always <5% for items with missing values) were imputed to the least risky and most frequent category when possible; relevant items were the recipient’s body mass index (BMI; 0.3%), duration of dialysis (0.8%), cardiovascular comorbidities (4.0%), and presence of diabetes (2.4%) as well as the donor’s eGFR (2.7%).

#### Propensity Score (PS) Matching

Because recipients were not randomly assigned to one of the two donor groups (LD or DBD), we followed the recommendations for emulating a target trial by constructing a PS to reduce selection bias [9]. The specification, emulation, and description of this target trial are described in Supplementary Table S1, Supplementary Text S1. The aim of the PS matching was to constitute a group of recipients with the same probability of receiving a kidney from LD and from DBD at the time of transplantation. We chose matching for the PS [11], that is, 1) estimating the probability of treatment (here the type of donation) from a multivariate logistic regression model according to recipients’ characteristics at KT, which may differ because of medical practices that vary by type of graft (in terms of age, sex, blood type, BMI, duration of dialysis, cardiovascular comorbidities, diabetes, and cPRA); and 2) using a greedy matching algorithm (caliper width 0.2, without replacement) to match one LD recipient to one DBD recipient with the same probability of LD treatment. The PS for the matching process included recipient age, duration of dialysis before transplantation, blood group, sex, and cPRA (Supplementary Table S2; Supplementary Figure S1).

Imbalance in each baseline covariate was defined as a standardized difference >0.2 and was computed for each recipient characteristic included in the PS, donor characteristic (age, hypertension, and eGFR at procurement), and KT characteristics (cold ischemia time, HLA A-B mismatches, HLA DR-DQ mismatches, and delta donor age–recipient age) to describe the potential differences between the two populations.

#### Association Between 1-Year eGFR and Type of Donor

To study the association between eGFR at 1 year and type of donor (LD or DBD), we used logistic generalized estimating equations taking into account matching. Confounders considered were recipient characteristics at KT (age, sex, blood type, BMI, duration of dialysis, cardiovascular comorbidities, ESRD cause, and immunization in three calculated cPRA classes). Because differences in delta age, HLA mismatches, and cold ischemia time are inherent in receiving a kidney graft from an LD or DBD, these variables were not included in our models. After stepwise selection, only variables with *p* < 0.05 were included in the multivariate final model. Furthermore, we performed an interaction test between donor type and donor age.

Because age is a major determinant of the interpretation of eGFR in both physiological conditions [12] and CKD [13], we conducted sensitivity analyses considering two additional eGFR thresholds at 1 year: 45 and 80 mL/min/1.73 m^2^.

We also analyzed eGFR as a continuous variable by recipient age and donor subgroup: DBD with standard criteria (DBD-SC) or expanded criteria (DBD-EC) and LD <60 years old (LD_<60y_) or ≥ 60 years old (LD_≥60y_); the latter considered an expanded-criteria LD. DBD-EC is defined by the American Organization of Transplantation and the United Network for Organ Sharing as DBD at age ≥ 60 years or 50–59 years with at least two of the following risk factors: donor hypertension, history of cerebrovascular accident, or terminal serum creatinine level ≥130 μmol/L [14]. Linear regressions by donor subgroup were used to investigate variation between the donor subgroups in the association between 1-year eGFR as a continuous variable and recipient age as a continuous variable.

We performed another sensitivity analysis, categorizing none of the continuous variables. In this analysis, continuous variables (age, BMI, and duration of dialysis) were transformed by using restricted cubic splines to estimate a new PS and to study the association between donation type and 1-year eGFR (Supplementary Text S2).

#### Graft and Recipient Survival by Type of Donor

Graft and recipient survival were studied at 4 years by type of donor in the matched cohort. Survival curves of graft and recipient groups were estimated by the Kaplan-Meier method and compared by the log-rank test.

Statistical analyses were performed with SAS Enterprise Guide 7.15 (SAS Institute, Cary, NC).

## Results

### Population Characteristics

The cohort included 1,496 LD and 8,097 DBD transplantations (Figure 1). Because of missing follow-up at 1 year, untimely follow-up, death during the first week post-KT, or missing eGFR, 591 DBD recipients and 106 LD recipients were excluded. The study included 1,390 LD and 7,506 DBD transplantations.

The LD donors were significantly younger than DBD donors (mean age 51 vs. 57 years; *p* < 0.0001), had higher eGFR at procurement (mean 95 vs. 73 mL/min/1.73 m^2^; *p* < 0.0001), and had hypertension less frequently (6.2% vs. 35.6%) (Table 1). As compared with DBD transplantations, LD transplantations had significantly shorter cold ischemia time (mean 3.6 vs. 15.9 h; *p* < 0.0001) and more HLA A-B mismatches (mean 1.6 vs. 1.2; *p* < 0.0001). The difference between donor age and recipient age (hereafter called delta age) was higher for LD than DBD recipients (mean 3 vs. 2 years; *p* = 0.02) (Table 1; Supplementary Table S3).

**TABLE 1:** Baseline characteristics of kidney transplantations by donation after brain death (DBD) and living donation (LD) for the overall study cohort and the matched cohort.

		Overall study cohort			Matched cohort		
		DBD 7,506 N (%)	LD 1,390 N (%)	Standardized difference	DBD 1,390 N (%)	LD 1,390 N (%)	Standardized difference
Recipient characteristics							
Recipient body mass index (kg/m^2^)	Underweight (<18.5 kg/m^2^)	289 (3.9)	69 (5)	0.16	70 (5)	69 (5)	<0.1
	Normal (18.5–24 kg/m^2^)	3,154 (42)	686 (49.4)		645 (46.4)	686 (49.4)	
	Overweight (≥25 kg/m^2^)	4,063 (54.1)	635 (45.7)		675 (48.6)	635 (45.7)	
Recipient sex	Male	4,762 (63.4)	934 (67.2)	<0.1	917 (66)	934 (67.2)	<0.1
	Female	2,744 (36.6)	456 (32.8)		473 (34)	456 (32.8)	
Recipient blood group	A	3,338 (44.5)	589 (42.4)	<0.1	616 (44.3)	589 (42.4)	<0.1
	AB	34 (4.6)	61 (4.4)		60 (4.3)	61 (4.4)	
	B	792 (10.6)	173 (12.4)		166 (11.9)	173 (12.4)	
	O	3,032 (40.4)	567 (40.8)		548 (39.4)	567 (40.8)	
Cause of ESRD	Chronic glomerulonephritis	1,547 (20.6)	389 (28)	0.27	320 (23)	389 (28)	0.15
	Diabetes (type I or II)	817 (10.9)	96 (6.9)		81 (5.8)	96 (6.9)	
	Kidney malformation or hereditary nephropathy	311 (4.1)	94 (6.8)		84 (6)	94 (6.8)	
	Chronic interstitial nephropathy	745 (9.9)	142 (10.2)		169 (12.2)	142 (10.2)	
	Nephroangiosclerosis	813 (10.8)	91 (6.6)		112 (8.1)	91 (6.6)	
	PKD	1,261 (16.8)	240 (17.3)		267 (19.2)	240 (17.3)	
	Others	2,012 (26.8)	338 (24.2)		357 (25.7)	338 (24.2)	
cPRA	0%	4,357 (58)	885 (63.7)	0.23	883 (63.5)	885 (63.7)	<0.1
	1%–84%	2,551 (34)	465 (33.5)		469 (33.7)	465 (33.5)	
	85%–100%	598 (8)	40 (2.9)		38 (2.7)	40 (2.9)	
Recipient age	18–39 years	1,292 (17.2)	422 (30.4)	0.45	418 (30.1)	422 (30.4)	<0.1
	40–54 years	2,175 (29)	485 (34.9)		488 (35.1)	485 (34.9)	
	55–59 years	938 (12.5)	162 (11.7)		165 (11.9)	162 (11.7)	
	≥60 years	3,101 (41.3)	321 (23.1)		319 (22.9)	321 (23.1)	
Cardiovascular comorbidities	No	6,352 (84.6)	1,250 (89.9)	0.16	1,245 (89.6)	1,250 (89.9)	<0.1
	Yes	1,154 (15.4)	140 (10.1)		145 (10.4)	140 (10.1)	
Recipient diabetes	No	6,034 (80.4)	1,201 (86.4)	0.16	1,212 (87.2)	1,201 (86.4)	<0.1
	Yes	1,472 (19.6)	189 (13.6)		178 (12.8)	189 (13.6)	
Duration of dialysis before transplantation	Preemptive transplantation	890 (11.9)	576 (41.4)	0.99	575 (41.4)	576 (41.4)	<0.1
	<3 years	3,636 (48.4)	718 (51.7)		719 (51.7)	718 (51.7)	
	≥3 years	2,980 (39.7)	96 (6.9)		96 (6.9)	96 (6.9)	
Time on waitlist	<1 year	2,263 (30.2)	908 (65.3)	0.81	544 (39.2)	908 (65.3)	0.54
	Between 1 and 3 years	3,171 (42.3)	394 (28.3)		657 (47.3)	394 (28.3)	
	>3 years	2,070 (27.6)	88 (6.3)		188 (13.5)	88 (6.3)	
Donor characteristics							
Donor age	<39 years	1,157 (15.4)	240 (17.3)	0.51	385 (27.7)	240 (17.3)	0.34
	40–54 years	1,927 (25.7)	605 (43.5)		427 (30.7)	605 (43.5)	
	55–59 years	841 (11.2)	192 (13.8)		154 (11.1)	192 (13.8)	
	≥60 years	3,581 (47.7)	353 (25.4)		424 (30.5)	353 (25.4)	
Donor hypertension	No	4,833 (64.4)	1,304 (93.8)	0.78	1,040 (74.8)	1,304 (93.8)	0.54
	Yes	2,673 (35.6)	86 (6.2)		350 (25.2)	86 (6.2)	
Donor eGFR at procurement	≥60 mL/min/1.73 m^2^	5,099 (67.9)	1,382 (99.4)	0.94	951 (68.4)	1,382 (99.4)	0.93
	<60 mL/min/1.73 m^2^	2,407 (32.1)	8 (0.6)		439 (31.6)	8 (0.6)	
Transplant characteristics							
*Cold ischemia time, hr*		15.9 (5.9)	3.6 (4.1)	2.43	15.4 (5.8)	3.6 (4.1)	2.35
*HLA A-B mismatches*		1.6 (0.5)	1.2 (0.6)	0.71	1.6 (0.5)	1.2 (0.6)	0.75
*HLA DR-DQ mismatches*		0.9 (0.6)	1 (0.7)	<0.1	0.9 (0.7)	1 (0.7)	0.12
Delta donor age–recipient age	<−3.5 years	1,277 (17)	412 (29.6)	0.39	287 (20.6)	412 (29.6)	0.34
	3.5–0 years	1,659 (22.1)	272 (19.6)		324 (23.3)	272 (19.6)	
	0–7 years	2,720 (36.2)	306 (22)		488 (35.1)	306 (22)	
	>7 years	1,850 (24.6)	400 (28.8)		291 (20.9)	400 (28.8)	

Note: Continuous variables are presented in italic as means (standard deviation); dichotomous variables as n (%).

BMI, body mass index; ESRD, end-stage renal disease; PKD, polycystic kidney disease; cPRA, calculated panel-reactive antibodies; eGFR, glomerular filtration rate estimated with the CKD-EPI formula (mL/min/1.73 m^2^); HLA, human leukocyte antigen; LD: living donation; DBD, donation after brain death.

As compared with DBD recipients, LD recipients were significantly younger (mean age 48 vs. 55 years; *p* < 0.0001) and more often male (67.2% vs. 63.4%), with fewer comorbidities (diabetes: 13.6% vs. 19.6%; cardiovascular comorbidities: 10.1% vs. 15.4%) and lower BMI (mean 25 vs. 26 kg/m^2^; *p* < 0.0001). The cause of ESRD for LD recipients was more often chronic glomerulonephritis (28% vs. 20.6% for DBD recipients) and they more frequently had cPRA of 0% (63.7% vs. 58%), a waitlist time <1 year (65.3% vs. 30.2%), and a preemptive transplantation (41.4% vs. 11.9%) than DBD recipients.

The matching procedure retained 1,390 LD and 1,390 DBD transplantations (Table 1). After matching on recipient characteristics, the standardized differences for recipient criteria (except waitlist time) were insignificant.

In the matched cohort, DBD donors were younger than LD donors for recipients aged 18–39 years (mean 32 vs. 47 years; *p* < 0.0001), whereas LD donors were younger than DBD donors for recipients aged ≥60 years (mean 58 vs. 73 years; *p* < 0.0001) (Supplementary Table S4).

### eGFR at 1 Year After KT

In the matched cohort, about 50% of both DBD-SC and LD_<60y_ recipients had an eGFR ≥60 mL/min/1.73 m^2^ at 1 year; this proportion decreased to 22% in older LD recipients and to 9% in DBD-EC recipients (Supplementary Figure S3). More precisely, at 1 year, the proportion of recipients with eGFR ≥80 mL/min/1.73 m^2^ was highest for DBD-SC recipients (19%); it was 13% for LD recipients <60 years and 2%–4% for LD recipients ≥60 years and DBD-EC recipients. Overall, 14% of DBD-EC recipients and 2%–4% of recipients of other types of donations died or had graft failure at 1 year.

**FIGURE 2:**
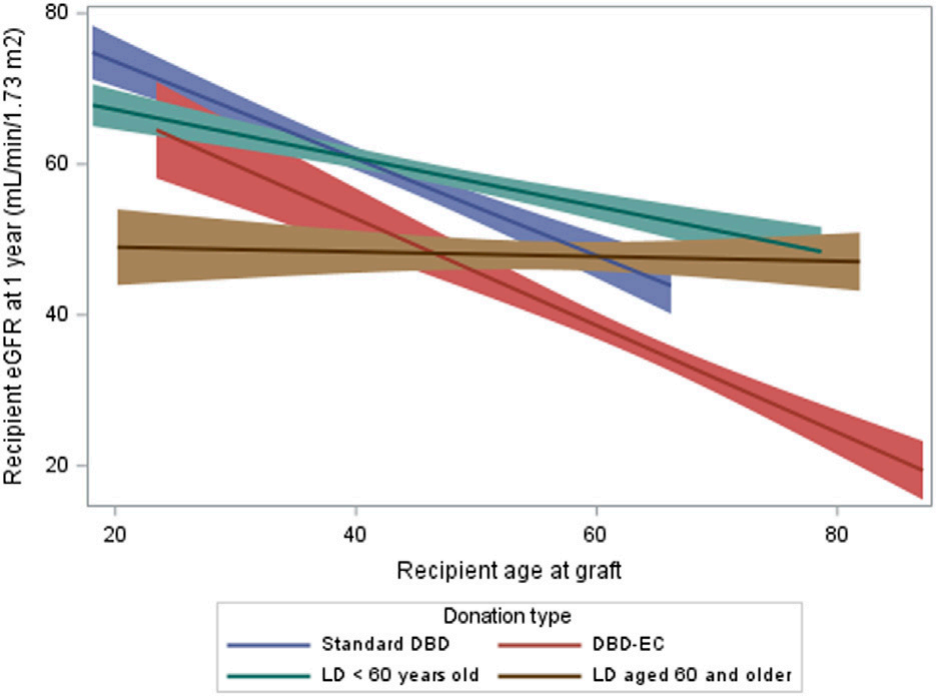
Recipient eGFR at 1 year by type of donation and recipient age in the matched cohort. eGFR, glomerular filtration rate estimated with the CKD-EPI formula (mL/min/1.73 m^2^); LD, living donation; DBD, donation after brain death.

Whatever the recipient’s age, the mean 1-year eGFR was about 50 mL/min/1.73 m^2^ for LD recipients aged ≥60 years (Figure 2). For recipients <40 years, 1-year eGFR was higher for DBD-SC recipients than other recipients. For recipients ≥60 years, 1-year eGFR was higher with all types of LD than with DBD.

**FIGURE 3:**
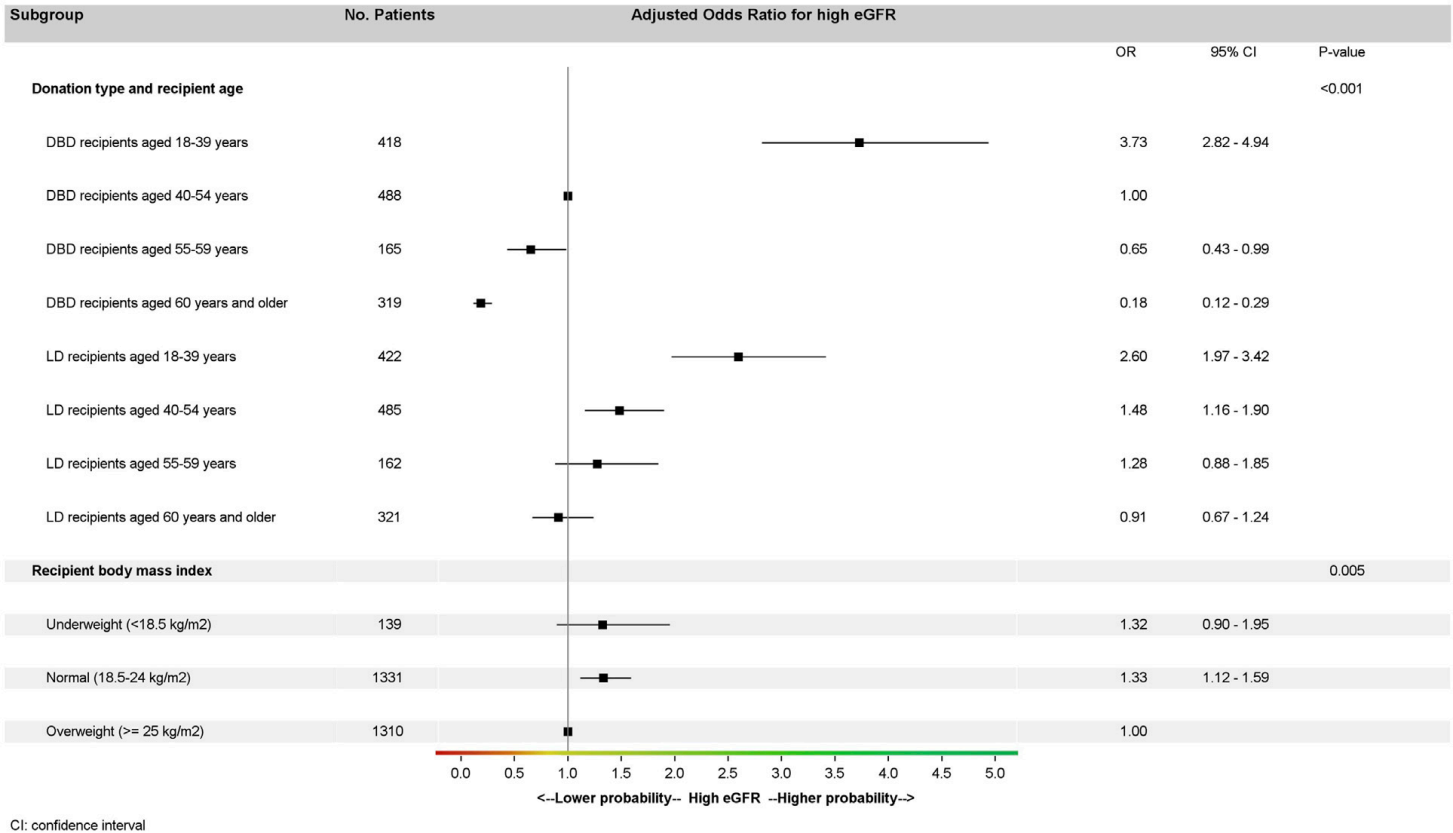
Multivariate analysis of high recipient eGFR at 1 year (*) in the matched cohort (generalized estimating equation model stratified by pairs). (*) High eGFR at 1-year is defined by eGFR ≥ 60 mL/min/1.73 m^2^; eGFR, glomerular filtration rate estimated with the CKD-EPI formula mL/min/1.73 m^2^; LD, living donation; DBD, donation after brain death.

In the multivariate model (Figure 3) of the matched cohort, high eGFR at 1 year (≥60 mL/min/1.73 m^2^) was more frequent for recipients with normal BMI than overweight recipients (OR: 1.33; 95% CI: 1.12–1.99, *p* = 0.005). We found an interaction between donation type and recipient age (*p* < 0.0001). For LD recipients aged 40–54 years versus same-aged DBD recipients, the adjusted odds ratio (aOR) for eGFR ≥60 mL/min/1.73 m^2^ was 1.48 (95% CI: 1.16–1.90). For DBD recipients ≥60 years, the aOR was 0.18 (95% CI: 0.12–0.29) versus DBD recipients 40–54 years but was 0.91 (95% CI: 0.67–1.24) versus LD recipients ≥60 years (i.e., 5.1 times higher).

**FIGURE 4:**
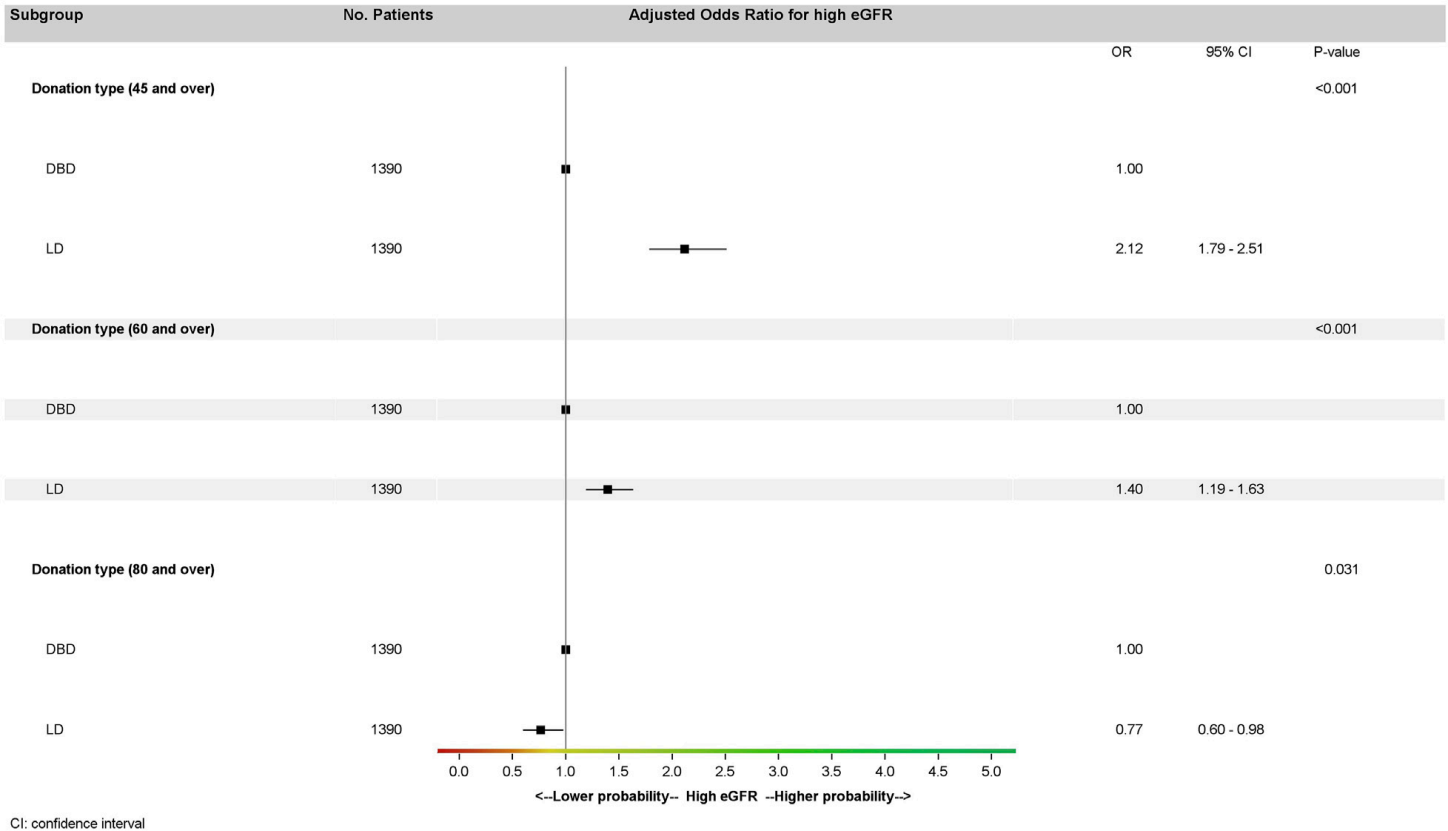
Multivariate analysis of eGFR at 1 year in the matched cohort (generalized estimating equation model stratified by pairs) according to several thresholds by donation type. Donor type odds ratios for high eGFR were adjusted on recipient age and recipient BMI for all three multivariate analyses. High eGFR at 1 year is defined by three different thresholds: eGFR ≥ 45 mL/min/1.73 m^2^, eGFR ≥ 60 mL/min/1.73 m^2^, and eGFR ≥ 80 mL/min/1.73 m^2^. eGFR, glomerular filtration rate estimated with the CKD-EPI formula mL/min/1.73 m^2^; LD, living donation; DBD, donation after brain death.

We performed sensitivity analyses on the matched cohort for different eGFR thresholds (45 mL/min/1.73 m^2^ and 60 and 80 mL/min/1.73 m^2^) (Figure 4) in the multivariate model. High eGFR was associated with type of donation regardless of the threshold considered but was more likely for LD recipients with a threshold at 45 mL/min/1.73 m^2^ (OR: 2.12; 95% CI: 1.79–2.51, *p* < 0.001) or 60 mL/min/1.73 m^2^ (OR: 1.40; 95% CI: 1.19–1.63, *p* < 0.001) and less likely for LD than DBD recipients with a threshold at 80 mL/min/1.73 m^2^ (OR: 0.77; 95% CI: 0.60–0.98, *p* = 0.03).

The sensitivity analysis with all continuous variables transformed by using restricted cubic splines revealed a significant interaction between recipient age and type of donor in the association with eGFR (*p* < 0.0001): eGFR was higher for recipients aged under 40 whatever the type of donor and that from the age of 55 eGFR was higher for LD than for DBD recipients (Supplementary Figure S4).

### Graft and Recipient Survival by Donor Subgroup

**FIGURE 5:**
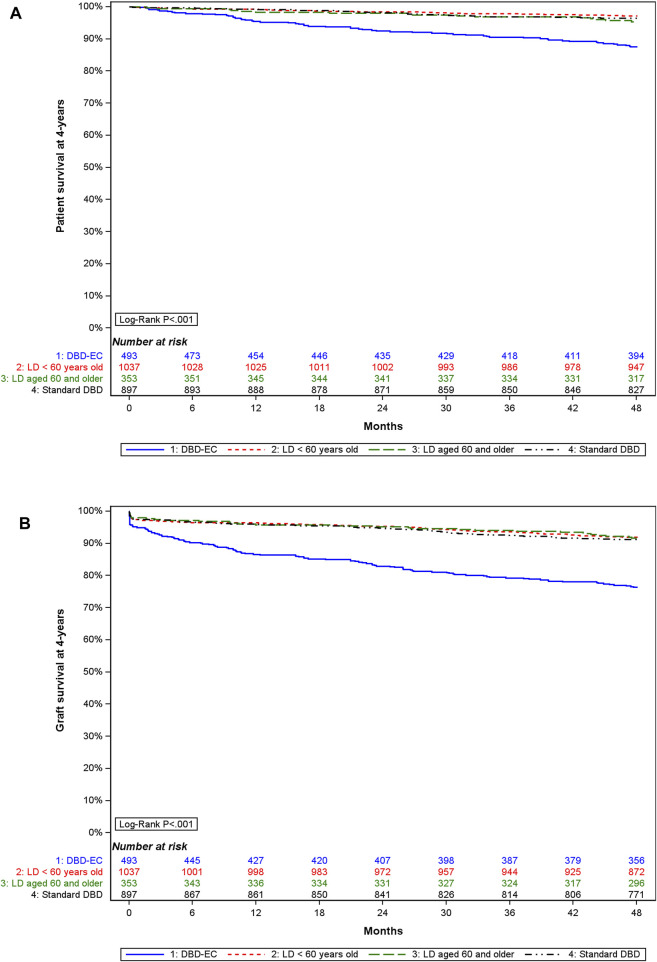
4-year **(A)** graft survival and **(B)** patient survival by type of donor in the matched cohort.

In the matched cohort, 4-year graft survival differed by donor subgroup (Figure 5): it was lowest with DBD-EC transplants (76.4%, 95% CI 72.4%–79.9%) versus DBD-SC transplants (91.2%, 95% CI 89.2%–92.9%), LD_<60y_ transplants (91.9%, 95% CI 90.0%–93.4%), and LD_≥60y_ transplants (91.6%, 95% CI 88.2%–94.1%). Similar results were found when analyzing 4-year patient survival (Figure 5).

## Discussion

LD transplantation is the preferred treatment for kidney failure, offering *a priori* superior patient and graft survival as compared with DBD transplantation. However, LD recipients are usually not comparable to DBD recipients, LD recipients typically being younger and having no or less pre-transplant dialysis duration. We attempted to emulate a target trial by creating a PS-matched cohort to investigate eGFR at 1 year after KT for DBD and LD recipients, ensuring comparability between the LD and DBD groups in recipient age, sex, blood group, pretransplant dialysis duration, and cPRA. The eGFR at 1-year post-transplant is widely considered the most relevant marker for predicting graft and patient survival after transplantation and is extensively used in randomized clinical trials [15]. At 1 year after KT, eGFR was significantly higher for LD than DBD recipients. Specifically, the OR for attaining an eGFR ≥45 mL/min/1.73 m^2^ was 2.12 times higher and an eGFR ≥60 mL/min/1.73 m^2^ was 1.40 times higher for LD than DBD recipients.

However, our study suggests that the superiority of LD over DBD in terms of eGFR is not consistent across all recipient age groups. Among recipients <40 years, the OR for an eGFR ≥60 mL/min/1.73 m^2^ did not significantly differ between DBD and LD recipients. For recipients ≥60 years, the OR for an eGFR ≥60 mL/min/1.73 m^2^ was 5 times higher for LD than DBD recipients (0.91 vs. 0.18 for younger LD recipients).

Several factors may explain this difference in eGFR between DBD and LD recipients based on recipient age. LD recipients <40 years frequently receive a kidney from an older LD donor, a situation that contrasts with the DBD allocation strategy, which often favors age matching. As a result, younger DBD recipients typically receive kidneys from young DBD donors [16]. This situation is supported by the greater delta donor age–recipient age in the LD than DBD group. In contrast, DBD recipients ≥60 years mainly receive kidneys from donors within the same age group, whereas LD recipients ≥60 years may receive kidneys from younger donors [16]. Additionally, DBD recipients ≥60 years may receive kidneys from DBD-EC donors, whereas LD recipients usually have few or no comorbidities. Hypertension and kidney aging are associated with a higher proportion of sclerotic glomeruli and nephron loss, leading to lower eGFR after KT [17–19]. Therefore, kidneys from an older LD donor may result in a lower eGFR than kidneys from a younger DBD donor. Our sensitivity analyses showed that a threshold eGFR of 80 mL/min/1.73 m^2^ was more common among DBD than LD recipients, who are often matched with younger donors. In contrast, at 1 year after KT, the mean eGFR was approximately 50 mL/min/1.73 m^2^ for recipients from LD donors ≥60 years old, regardless of recipient age.

At 1 year after KT, donor age has been found correlated with renal function as well as long-term graft and patient survival [20–24]. Lim et al. [25] reported an association between donor age, 1-year eGFR, and overall graft loss. However, eGFR <60 mL/min/1.73 m^2^ at 1 year with a kidney from an older LD is expected and should not preclude the selection of an LD donor as suitable [26, 27].

In our matched cohort, the analysis of graft and patient survival at 4 years post-KT highlights the inferior outcomes of transplantation from a DBD-EC. Indeed, at 4 years, the graft survival was approximately 15% lower for recipients of a DBD-EC than for other recipients. The graft acceptance criteria are more extensive in France than in the United States [28, 29]. However, graft survival from a DBD-EC donor in France is comparable to literature data and is considered satisfactory as compared with dialysis maintenance [17, 30]. In older patients, long-term results were found better with LD than DBD-SC or DBD-EC and suggested the use of LD transplants in older patients whenever possible [18]. However, in Japan, the age of the oldest LD is high (70–89 years) and outcomes were found to be poor in terms of graft survival and eGFR for older recipients from very old LD donors [31]. In the same way, from UK registry data, all-cause mortality was greater for recipients of older LD (donor age ≥70 years) than DBD-SC and was equivalent to that for DBD-EC recipients [7].

Our study has some limitations. It was conducted in France, where national practices for DBD allocation and organ acceptance differ from those in other countries. Different allocation scores and stricter kidney acceptance criteria in other regions may yield different results between DBD and LD recipients. The particular strength of our work lies in its methodology, effectively used in other studies [32, 33], emulating a target trial that is not feasible in the real world. This method allows for defining all the conditions required for the target trial and precisely describing the deviations from it. However, some important factors may not have been taken into account in our PS. Furthermore, we excluded recipients without timely follow-up or who died in the first week before matching. Because they represented less than 10% of the target population and their distribution was not different between LD and DBD recipients, we considered the resulting selection bias to be negligible.

A second limitation is the lack of long-term follow-up. Nevertheless, we needed to begin inclusions in 2015 to have both a homogeneous donor population (sharp increase in age of donors since 2000) and the same kidney allocation system throughout the cohort (the current kidney allocation system was implemented in 2015).

A third limitation could be our choice to conduct stepwise regressions based on *p*-value and involving multiple comparisons; we opted for this choice because of our sample tail that allowed for a high number of degrees of freedom. Furthermore, we opted to categorize all our continuous variables, which implies loss of information and precision [34, 35]. We made this choice after discussion with clinicians who preferred having information directly usable with the references they used in practice. To test the robustness of our results, we conducted a sensitivity analysis performed with Harrell’s recommendations and found consistent results.

A final limitation is the use of eGFR at 1 year after KT as a single surrogate marker of graft outcome. Indeed, predicted graft survival based on this surrogate marker is correlated with observed graft survival [36] and this parameter is used in studies testing new treatments [37]. However, graft injuries may develop slowly over time and eGFR at 1 year fails to capture ongoing disease process [38]. In our matched cohort on recipient characteristics, we tested eGFR at 1 year after KT depending on donor type stratified by age. So, we discussed eGFR at 1 year as a marker potentially reflecting nephron loss more than nephron injury that leads to graft failure.

Our study showed that older recipients derive significant benefits from LD transplants, which emphasizes the importance of evaluating living donors ≥60 years old. Conversely, transplants from DBD donors can yield good outcomes for young adult recipients, provided that there is a suitable age and HLA match, along with prompt access to transplantation. Of note, our matched cohort reflects a notably short pre-transplant dialysis period, a characteristic often associated with LD transplants. Paired-exchange programs offer a viable avenue to explore improved age matching, particularly with a significant age gap between the donor and recipient. However, KT for compatible donor-recipient pairs seeking a better match in terms of age should not be delayed too long, and the search for a better match should be carried out early.

Our study is the first published research to use an emulated target trial to compare LD and DBD recipients. These findings offer valuable insights for healthcare professionals, empowering them to make well-informed decisions regarding the suitability of different donor types for specific recipients.

## Data Availability

The data analyzed in this study is subject to the following licenses/restrictions: In accordance with French law, research studies based on Cristal national registry are part of transplant assessment and do not require additional institutional review board approval. The database has been reported to the French National Commission on Computing and Liberty. Requests to access these datasets should be directed to nicolas.chatauret@biomedecine.fr.

## Abbreviations

LD, living donation; DBD, donation after brain death; DBD-EC, donation after brain death with expanded criteria; KT, kidney transplantation; eGFR, estimated glomerular filtration rate from plasma creatinine; HLA, human leukocyte antigen; cPRA, calculated panel-reactive antibodies; BMI, body mass index; ESRD, end-stage renal disease.
