# Supplementary material for: Comparison of Kidney Graft Function and Survival in an Emulated Trial With Living Donors and Brain-Dead Donors
Source: Transpl Int. 2024 Aug 29;37:13208. doi: 10.3389/ti.2024.13208 (PMC11391114; doi:10.3389/ti.2024.13208)
Supplement: Supplementary file 1 [file DataSheet1.docx]

**Supplementary Appendix**

This appendix has been provided by the authors to give readers additional information about their work. This supplement has additional information on methods and results.

Supplement to: E. Savoye, G. Santin, F. Kerbaul et al. Comparison of kidney graft function after living and deceased donations across recipient and donor ages: an observational analysis emulating a target trial

**Table of contents**

[Supplementary list S1. Cristal registry study group 2](#_Toc173747668)

[Supplementary Table S1. Specification and emulation of a target trial evaluating the comparative effect of a living donation (LD) kidney graft and a donation after brain death (DBD) kidney graft by using observational data from the CRISTAL database (2015-2018) 5](#_Toc173747669)

[Supplementary text S1. Description of the emulation target trial 6](#_Toc173747670)

[Supplementary Table S2. Propensity score used for matched procedure between recipients receiving a kidney from a living donor or from a donor after brain death 9](#_Toc173747671)

[Supplementary Figure S1. Jittered plot showing matched and unmatched observations as well as their distribution on propensity score values 10](#_Toc173747672)

[Supplementary Figure S2. Box plot of donor age according to recipient age and type of donation in the matched cohort 13](#_Toc173747673)

[Supplementary Figure S3. Distribution of recipient eGFR at 1 year in the matched cohort by type of donation 13](#_Toc173747674)

[Supplementary Table S4. Description of continuous variables studied 14](#_Toc173747675)

[Supplementary text S2. Final sensitivity analysis method 15](#_Toc173747676)

[Supplementary Figure S4. Prediction from new_model of eGFR (mL/min/1.73 m2) at 1 year according to recipient age at graft in the matched cohort Match_PS2 16](#_Toc173747677)

[Capsule Sentence Summary 17](#_Toc173747678)

[Older recipients benefit more from living donor kidney transplants compared to younger recipients, as demonstrated in an emulated trial involving living and brain-dead donor. 17](#_Toc173747679)

Supplementary list S1. Cristal registry study group

**Cristal registry study group:** ABBO Olivier CHU Toulouse, 36760, Pediatric Surgery, Toulouse, Occitanie, France; ABDO Nicolas Service d'urologie et de transplantation rénale, CHU de Montpellier, 34295 Montpellier cedex 5, France; ALBANO Laetitia Unité de Transplantation Rénale, Hôpital Pasteur 2, CHU Nice, Nice, France; ALEZRA Eric, Department of Urology and Kidney Transplantation, University Hospital of Bordeaux, Bordeaux, France; AMMAR Hatem Department Anesthesiology, Necker Hospital, Assistance Publique-Hôpitaux de Paris, Paris, France; ANGLICHEAU Dany Department of Nephrology and Kidney Transplantation, Necker Hospital, Assistance Publique-Hôpitaux de Paris, Paris, France.; ARMAND Aurore, Emergency Department, Angers University Hospital, UNIV Angers, Angers, France.; ASSATOURIAN Savva Department of Anesthesia and Critical Care, Magellan Medico-Surgical Centre, CHU Bordeaux Bordeaux, France; BAUDOUIN Véronique Assistance Publique des Hôpitaux de Paris (AP-HP), Hôpital Robert-Debré, Département de Néphrologie pédiatrique, Paris, France; BEN AHMED Sabrina Service de Chirurgie Vasculaire et Cardiovasculaire, CHU Saint-Etienne, Saint-Etienne, France; BERTRAND Dominique Nephrology-Kidney Transplant Unit, Rouen University Hospital, Rouen, France; BESSEDE Thomas Hôpital Kremlin Bicetre, Urology and Transplantation, Paris, Paris, France; BLANCHO Gilles Institut de Transplantation Urologie Néphrologie (ITUN), CHU Nantes, Nantes, France; BOUTIN Jean Michel Department of Urology CHU de Tours Tours France; BRANCHEREAU Julien CHU de Nantes, Urology and Transplantation, Nantes, Nantes, France.; BRUYERE Franck, CHRU Bretonneau, Departments of Pathology and Urology, Tours, France; BUCHLER Matthias Service de Néphrologie-Hypertension Artérielle, Dialyses, Transplantation Rénale, CHRU de Tours, Tours France; BURON Fanny Department of Nephrology, Ed. Herriot Hospital, Lyon, France; CAILLARD Sophie Department of Nephrology and Kidney Transplantation, University Hospital of Strasbourg, Strasbourg, France; CHAKFE Nabil EA 3072, Institut de Physiologie, Faculté de Médecine de Strasbourg, 67000 Strasbourg, France; Service de Chirurgie Vasculaire et Transplantation Rénale, Hôpitaux Universitaires de Strasbourg, 67000 Strasbourg, France; CHAMPY Cécile AP-HP, Hôpitaux Universitaires Henri Mondor, Service d'urologie, Créteil, France; CHAUVET Romain, Service de chirurgie vasculaire et de médecine vasculaire, CHU Limoges, Limoges, France; CLAISSE Guillaume Nephrology, Dialysis and Renal Transplantation Department, University Hospital of Saint Etienne, Saint Etienne, France; CLAVE Stéphanie Pediatric Nephrology Unit, Marseille University Hospital, Marseille, France; CORMIER Luc Department of Urology, CHU Dijon France; COUZI Lionel Department of Nephrology, Transplantation, Dialysis and Apheresis, CHU Bordeaux, Bordeaux, France; DANTAL Jacques Institut de Transplantation Urologie Néphrologie (ITUN), CHU Nantes, Nantes, France; DE SOUSA Philippe Service d’urologie-transplantation, université de Picardie-Jules-Verne, CHU Amiens-Picardie, avenue René-Laennec, 80054 Amiens cedex 1, France; DEL BELLO Arnaud Département de Néphrologie et Transplantation d'Organes, CHU Rangueil, Toulouse, France; DELAPORTE Véronique Department of Urology and Kidney Transplantation, Assistance Publique-Hôpitaux de Marseille (APHM-HUM), La Conception University Hospital, 147 Boulevard Baille, 13005, Marseille, France; DELOFFRE Isabelle Coordination des prélèvements d’organes et de tissus - Pôle Anesthésie - Soins critiques - CHPOT - Hôpital Pasteur 2, CHU de Nice, Nice, France; DELPECH Pierre-Olivier Service d'urologie, CHU de Poitiers, 2, rue de la Milétrie, 86000 Poitiers, France; DEVEZE Eva, Department of Vascular Surgery, University Hospital of Angers, Angers, France; DORE Julien Department of Nephrology and Kidney Transplantation, Necker Hospital, Assistance Publique-Hôpitaux de Paris, Paris, France; DUCOS Guillaume CHU de Toulouse, Service de réanimation polyvalente, Toulouse F-31000, France; DUNAND Olivier Service de Néphrologie Pédiatrique, Centre Hospitalier Universitaire Felix Guyon, Saint Denis, France; DURAND Matthieu Urology, Andrology, Renal Transplant Unit, Hôpital Pasteur 2, Nice University Hospital, Nice, France; DUVEAU Agnès Department of Nephrology and Transplantation, University of Angers, Angers, France; ECOTIERE Laure Service de Néphrologie, Hémodialyse et Transplantation rénale, Centre Hospitalier Universitaire de Poitiers, Poitiers, France; EPINAT Magali Neurovascular Unit, Centre Hospitalier Universitaire de Saint-Etienne, Saint-Etienne, France; ESCARAVAGE Laurence Department of Anesthesiology, University Hospital of Clermont-Ferrand, Clermont-Ferrand 63000, France; ESPOSITO Laure Department of Nephrology and Organ Transplantation, Toulouse Rangueil University Hospital, 31073 Toulouse, France; ETHUIN Frédéric Côte de Nacre University Hospital, Surgical Intensive Care, Caen, France; FEDOU Anne-Laure, Medical-Surgical Intensive Care Unit, Dupuytren Teaching Hospital, 87000, Limoges, France; FIARD Gaelle Service d’Urologie et de la Transplantation rénale  CHU Grenoble-Alpes, Grenoble , France.; FILA Marc Pediatric Nephrology Unit, CHU Arnaud de Villeneuve-Université de Montpellier, Montpellier, France; FLAHAUT Gauthier Service de Néphrologie. université de Picardie-Jules-Verne, CHU Amiens-Picardie, avenue René-Laennec, 80054 Amiens cedex 1, France; FOURNIER Catherine Department of Nephrology and Kidney Transplantation, Necker Hospital, Assistance Publique-Hôpitaux de Paris, Paris, France; FRANCOIS Hélène , Department of Nephrology and Renal Transplantation, Assistance Publique-Hôpitaux de Paris, Hôpital Tenon, Paris, France; FRANQUET Quentin       Service d’Urologie et de la Transplantation rénale  CHU Grenoble-Alpes, Grenoble , France. ; FRIMAT Luc Service de néphrologie, Hôpitaux de Brabois, Centre hospitalier régional universitaire de Nancy, rue du Morvan, 54511 Vandœuvre-lès-Nancy, France; FRONTCZACK Alexandre CHU de Besançon, urologie, 25030 Besançon cedex, France; GARAIX Florentine Service de Pédiatrie Multidisciplinaire, Hôpital de La Timone, Marseille, France; GARROUSTE Cyril Service de néphrologie, dialyse et transplantation, CHU de Gabriel-Montpied, 58, rue Montalembert, BP 69, 63000 Clermont-Ferrand cedex, France; GIRAL Magali, CHU Nantes, Nantes Université, Institut de Transplantation Urologie Néphrologie, Nantes, France; GOUJON Anna Department of Urology, Rennes University Hospital, Rennes, France; GREGOIRE Hélène Pôle d’Anesthésie-Réanimation, Centre Hospitalier Universitaire de Nancy, Nancy, France; GRIMBERT Philippe Department of Nephrology, Assistance Publique Hopitaux de Paris (APHP), Creteil, France.; GUIDICELLI Gwenda-line CHU de Pellegrin, laboratoire immunologie et immunogénétique, place Amélie-Raba-Leon, 33076 Bordeaux cedex, France; HENG Anne-elisabeth Service de néphrologie, dialyse et transplantation, CHU de Gabriel-Montpied, 58, rue Montalembert, BP 69, 63000 Clermont-Ferrand cedex, France; HERTIG Alexandre Department of Nephrology, Hospital Foch, 40, Rue Worth, 92150 Suresnes, France; IBORRA François Service d'urologie et transplantation rénale, CHU Montpellier, Montpellier, France; INGELS Alexandre AP-HP, Hôpitaux Universitaires Henri Mondor, Service d'urologie, Créteil, France; JACOBS Julie Nephrology-Kidney Transplant Unit, Rouen University Hospital, Rouen, France; JALAL-EDDINE Arwa Department of Nephrology, Hospital Foch, 40, Rue Worth, 92150 Suresnes, France; JANBON Bénédicte Service de Néphrologie, Hémodialyse , Aphérèses et Transplantation Rénale, CHU Grenoble-Alpes , Grenoble , France. ; KAMAR Nassim Department of Nephrology and Organ Transplantation , Toulouse University Hospital Center, Toulouse, France; KARAM Georges Department of Urology and Transplantation Surgery, Nantes University Hospital, Nantes, France; KERFORNE Thomas Service d'Anesthésie et de Réanimation, CHU de Poitiers, Poitiers, France; LAURENT Charlotte Nephrology-Kidney Transplant Unit, Rouen University Hospital, Rouen, France; LE MEUR Yannick, Nephrology Department, University Hospital, Brest, France; LE QUINTREC Moglie Service de Néphrologie CHU de Montpellier Montpellier France; LEBRET Thierry Service d'Urologie, Hôpital Foch, Suresnes, France.; LEGENDRE Christophe Department of Nephrology and Kidney Transplantation, Necker Hospital, Assistance Publique-Hôpitaux de Paris, Paris, France.; LEGRIS Tristan AP-HM, CHU Conception, Centre de Néphrologie et Transplantation Rénale, 147 Bd Baille, 13005, Marseille, France; LEJAY Anne EA 3072, Institut de Physiologie, Faculté de Médecine de Strasbourg, 67000 Strasbourg, France; Service de Chirurgie Vasculaire et Transplantation Rénale, Hôpitaux Universitaires de Strasbourg, 67000 Strasbourg, France; MAILLARD Nicolas Service de Néphrologie, Dialyse et Transplantation Rénale, Hôpital Nord, CHU de Saint-Etienne, France; MALVEZZI Paolo Service de Néphrologie, Hémodialyse , Aphérèses et Transplantation Rénale, CHU Grenoble-Alpes , Grenoble , France.; MARTIN LEFEVRE Laurent Service de Médecine Intensive Réanimation, Centre Hospitalier Departemental Les Oudairies, La Roche-sur-Yon, Pays de la Loire, France; MATIGNON Marie Department of Nephrology and Renal Transplantation, Assistance Publique-Hôpitaux de Paris, Institut Francilien de Recherche en Néphrologie et Transplantation IFRNT, Groupe Hospitalier Henri-Mondor/Albert-Chenevier, Université Paris-Est-Créteil, Département Hospitalo-Universitaire, Virus-Immunité-Cancer, Institut Mondor de Recherche Biomédicale, Equipe 21, INSERM U 955, Créteil, France.; MEJEAN Arnaud  Georges Pompidou European Hospital, Assistance Publique Hôpitaux de Paris, Department of Urology, Paris, France; MICHEL Jean-Luc Department of Pediatric Surgery, CHU de La Réunion, Saint-Denis, France; MILLET Clémentine Department of Urology, University of Clermont-Ferrand, Clermont-Ferrand, France; MOAL Valérie Centre de Néphrologie et Transplantation Rénale, Aix Marseille Université, Hôpitaux Universitaires de Marseille, Hôpital Conception, Marseille, France; MOIA Alessia Service de Chirurgie Vasculaire et Cardiovasculaire, CHU Saint-Etienne, Saint-Etienne, France; MORIN Marie-Pascale Service de Néphrologie, Hôpital de Pontchaillou, CHU de Rennes, Rennes, France.; MOULIN Bruno Department of Nephrology and Kidney Transplantation, University Hospital of Strasbourg, Strasbourg, France; MURCIA Ghislaine Nephrology-Kidney Transplant Unit, Rouen University Hospital, Rouen, France; OUALI Nacera Department of Nephrology, Assistance Publique-Hôpitaux de Paris, Tenon Hospital, Sorbonne Universite, Paris, France; OUENDO Martial UR7516 CHIMERE (Chirurgie, imagerie et régénération tissulaire de l'extrémité céphalique), université de Picardie Jules Verne, Amiens, France - Service d'anesthésie réanimation, CHU Amiens, 80054 Amiens, France; PADILLA Marc Service de Néphrologie, Dialyse, Aphérèses et Transplantation rénale Unité de Coordination des Prélèvements d'Organes et de Tissus, CHU Grenoble-Alpes , Grenoble , France.; PARIER Bastien Department of Urology, Hôpital Bicêtre, Université Paris Saclay, APHP, Le Kremlin-Bicêtre, France; PAYE-JAOUEN Annabel Department of Pediatric Urology, Robert Debré Hospital, APHP, Paris, France; PERNIN Vincent Kidney Transplant Unit, Nephrology Department. Montpellier University Hospital, Montpellier, France; PIETAK Michel Department of Urology and Renal Transplantation, Hôpital Européen Georges Pompidou, AP-HP, 20 rue Leblanc, 75015, Paris, France; PRIN Sébastien Department of Intensive Care, Dijon Bourgogne University Hospital, Dijon, France.; PRUDHOMME Thomas Department of Urology, Rangueil University Hospital, Toulouse, France; RENAULT Anne Medical ICU, La Cavale Blanche University Hospital, Brest, France; REROLLE Jean-Philippe, Department of Nephrology and Transplantation, University of Limoges, Limoges, France; RIOU Philippe Sorbonne Université, UMR-S INSERM 1166, IHU ICAN and coordination des prélèvements d’organes etr de tissus, hôpital Pitié-Salpêtrière, Assistance Publique-Hôpitaux de Paris, Paris, France; ROGIER Julien Organ Procurement Unit CHU Pessac France; ROSTAING Lionel Service de Néphrologie, Hémodialyse , Aphérèses et Transplantation Rénale, CHU Grenoble-Alpes , Grenoble , France. ; RUSCIO Laura Anesthésie Réanimation, Hopital Bicetre, Le Kremlin-Bicetre, Île-de-France, France; SALLUSTO Federico Département d'urologie, de transplantation rénale et d'andrologie, CHU de Toulouse, Toulouse, France; SI LARBI Anne Gaelle Medical and Surgical Intensive Care Unit, Foch Hospital, Suresnes, France; SIMON Thomas Department of Pediatric Internal Medicine, Rheumatology and Nephrology, Centre De Référence Des Maladies Rénales Rares du Sud-Ouest, SoRare, Toulouse University Hospital, Toulouse, France; SNANOUDJ Renaud Department of Nephrology, Hospital Bicêtre, 94220 Le Kremlin Bicêtre, France; SOOROJEBALLY Yanish Service d'Urologie, Hôpital Foch, Suresnes, France.; TERRIER Nicolas   Service d’Urologie et de la Transplantation rénale  CHU Grenoble-Alpes, Grenoble , France.; THIERRY Antoine FHU SUPORT, Limoges, F-87000, France; CHU Poitiers, Department of Nephrology, Dialysis and Transplantation, F-86000 Poitiers, France; THURET Rodolphe Service d'urologie et de transplantation rénale, CHU de Montpellier, 34295 Montpellier cedex 5, France; TIBI Brannwel Department of Urology, Andrology and Renal Transplant, Pasteur II University Hospital, 30 Avenue Romaine, 06001, Nice, France; TILLOU Xavier CHU de Caen, Urology and Transplantation, Caen Calvados, France; TIMSIT Marc-Olivier Department of Urology and Renal Transplantation, Hôpital Européen Georges Pompidou, AP-HP, 20 rue Leblanc, 75015, Paris, France; TINEL Claire Department of Nephrology and Renal Transplantation, François-Mitterrand University Hospital, 14 Rue Paul Gaffarel, BP 77908, 21079 Dijon, France.; UHL Marine Service d’urologie-transplantation, université de Picardie-Jules-Verne, CHU Amiens-Picardie, avenue René-Laennec, 80054 Amiens cedex 1, France; VACHIERY LAHAYE Florence Donation and Transplantation Coordination Unit, CHU Montpellier, Montpellier University Medical Center, Montpellier, France; VENHARD Jean Christophe Department of Anesthesia and Critical Care Medicine, French Society of Organ Procurement Medicine, University Hospital, Tours, France; WAECKEL Thibaut Department of Urology, Caen University Hospital, Caen, France; WIRAMUS Sandrine Aix Marseille Université, Hôpitaux Universitaires de Marseille, Service d'Anesthésie et de Réanimation, Hôpital de la Conception, 13005 Marseille, France.; WOLF Philippe Department of Vascular Surgery and Kidney Transplantation, University Hospital of Strasbourg, Strasbourg, France

Supplementary Table S1. Specification and emulation of a target trial evaluating the comparative effect of a living donation (LD) kidney graft and a donation after brain death (DBD) kidney graft by using observational data from the CRISTAL database (2015-2018)

| **Protocol component** | **Target trial specification** | **Target trial emulation** |
| --- | --- | --- |
| **Eligibility criteria** | Patients hospitalized in metropolitan France in 2015 through 2018 for a first single-organ adult kidney transplants without incompatibility for HLA type or ABO blood group, who were going to receive a transplant from an LD or a DBD | A hospitalized patient for a kidney graft was identified as a kidney graft recipient. Same as for the target trial except exclusion of: * patients without follow-up at 1-year * patients with untimely follow-up  * deaths in the first week * eGFR at 1 year missing |
| **Treatment strategy** | Receive a kidney graft from an LD or from a DBD. This implies that we consider that the different allocation rules of these grafts are included in the treatment: in other words, differences between donor age and recipient age (hereafter "delta age"), HLA mismatches, and cold ischemia time are considered to be inherent in receiving a kidney graft from an LD or from a DBD. | Same as for the target trial |
| **Treatment assignment** | Recipients were randomly assigned at baseline to one of the treatments (kidney from a LD or a kidney from a DBD) within strata defined by patient characteristics at baseline (age, sex, blood type, BMI, duration of dialysis, cardiovascular comorbidities, diabetes, cPRA) to take into account the known differences in medical practices in the allocation of a graft from an LD or a DBD. | We assumed random assignment after a 1:1 matching of patients who were transplanted with a kidney from an LD or a kidney from a DBD. Factors used for matching were those used for stratified randomization in the target trial. |
| **Outcome** | eGFR estimated with the CKD-EPI equation at 1 year after transplantation, dichotomized at 60 ml/min/1.73 m^2^ | Same as for the target trial |
| **Follow-up** | For each patient, follow-up starts on the day of graft and ends 1 year after baseline | For each patient, follow-up starts on the day of graft and ends 1-year after baseline. Follow-up was considered exploitable if it was done between 9 and 20 months after the transplant |
| **Causal contrasts** | Intention to treat effect | Intention to treat effect |
| **Statistical analysis** | Matched logistic regression | Analyses of logistic generalized estimating equations were conducted with matching taken into account. Confounders included in the model were recipient characteristics at graft: age, sex, blood type, BMI, duration of dialysis, cardiovascular comorbidities, diabetes, cause of ESKD, immunization (in 3 classes of cPRA). |

BMI: body mass index; cPRA: calculated panel-reactive antibodies; eGFR: estimated glomerular filtration rate; ESKD: end-stage kidney disease

Supplementary text S1. Description of the emulation target trial

To describe how to emulate a target trial with our observational data, we first designed the ideal randomized trial that we would have conducted if it were possible to investigate this question of interest and then defined the methodology used to emulate our target trial (Supplementary Table S1).

**Target trial specification**

*Eligibility criteria* included patients hospitalized in metropolitan France in 2015 to 2018 for a first adult single-organ kidney transplantation (KT) without incompatibility for HLA type or ABO blood group, that is, likely to receive a transplant from a living donation (LD) or donation after brain death (DBD).

*Treatments of interest* were KTs from an LD or DBD. Among other things, this means that we consider that the different allocation rules of these grafts are included in the treatment: differences between donor age and recipient age (hereafter called delta age), HLA mismatches, and warm or cold ischemia time are considered inherent in KT by LD or DBD.

Recipients were randomly assigned at baseline to one of the treatments within strata defined by each patient's baseline characteristics (age, sex, blood type, body mass index [BMI], duration of dialysis, cardiovascular comorbidities, diabetes, calculated panel-reactive antibodies [cPRA]) to take into account the known differences in medical practices related to the allocation of a graft from each donation type.

*The outcome of interest* was the eGFR, estimated with the CKD-EPI equation^11^ at 1 year after KT. Kidney function was considered high in recipients with eGFR ≥ 60 mL/min/1.73m^2^ at 1 year and low in those with eGFR < 60 mL/min/1.73m^2^. Recipients who died or experienced graft failure < 1 year after the KT were classified in the group with eGFR < 60 mL/min/1.73 m^2^.

**Target trial emulation**

We used the CRISTAL database *to emulate* our target trial. The French national transplant registry (CRISTAL, Agence de la Biomédicine, Saint-Denis, France) prospectively collects demographic, clinical, and laboratory data for all organ transplant recipients and donors in France as well as transplant outcomes. Data are recorded at registration (placement on a transplantation wait list), procurement, transplantation, and annually thereafter. Data collection is mandatory, and research technicians double-check its completeness and accuracy. In accordance with French law, research studies based on this national registry are part of transplant assessment and do not require additional institutional review board approval. The database has been reported to the French National Commission on Computing and Liberty.

*Eligibility criteria* were the same as for the target trial except for the exclusion of recipients who died in the first week after the transplant or with outlying or untimely follow-up durations (< 9 months or > 21 months after the transplant) or with an eGFR at 1 year > 150 mL/min/1.73 m² (data were missing for 8% of LD and 7% of DBD transplants, detailed in Figure 1).

*Outcome and treatment of interest were* the same as for the target trial.

*Treatment assignment* was assumed to be random after matching. More precisely, because recipients were not randomly assigned to one of the two donation groups (LD or DBD), a propensity score (PS) was constructed to reduce the selection bias so that the study was as close as possible to a randomized trial.^10^ The PS is a balancing score that enables similarity between LD and DBD donors in the distribution of the variables in the PS model and thus the creation of two comparable recipient groups.

We chose matching for the PS, ^11,12^ that is, 1) estimating the probability of treatment (here the type of donors) from a multivariate logistic regression model according to recipients' characteristics at KT, which may differ because of medical practices that vary by the type of graft (age, sex, blood type, BMI, duration of dialysis, cardiovascular comorbidities, diabetes, and cPRA), and 2) using a greedy matching algorithm (caliper width at 0.2, without replacement, to match one recipient of a kidney LD and one recipient of a kidney DBD to create two comparable recipient groups.

Supplementary Table S2. Propensity score used for matched procedure between recipients receiving a kidney from a living donor or from a donor after brain death

| **Parameter** |  | **Estimate** | **Odds ratio** | **95% Wald Confidence limits** |
| --- | --- | --- | --- | --- |
| Intercept |  | -2.46 |  |  |
| Recipient age (ref 60 years and more) | 18-39 years | 1.03 | 2.81 | 2.37-3.33 |
|  | 40-54 years | 0.67 | 1.95 | 1.66-2.29 |
|  | 55-59 years | 0.58 | 1.78 | 1.44-2.22 |
| Duration of dialysis before transplantation (ref <3 years) | 3 years and more | -1.85 | 0.16 | 0.13-0.2 |
|  | Preemptive transplantation | 1.23 | 3.43 | 2.99-3.93 |
| Recipient blood group (ref A) | AB | 0.08 | 1.09 | 0.8-1.47 |
|  | B | 0.53 | 1.69 | 1.38-2.08 |
|  | O | 0.36 | 1.44 | 1.25-1.65 |
| Sex (ref Female) | Male | 0.18 | 1.2 | 1.05-1.37 |
| cPRA (ref 0%) | 1-84% | 0.07 | 1.07 | 0.93-1.22 |
|  | 85-100% | -1.04 | 0.35 | 0.25-0.5 |

cPRA: calculated panel reactive antibodies

Supplementary Figure S1. Jittered plot showing matched and unmatched observations as well as their distribution on propensity score values


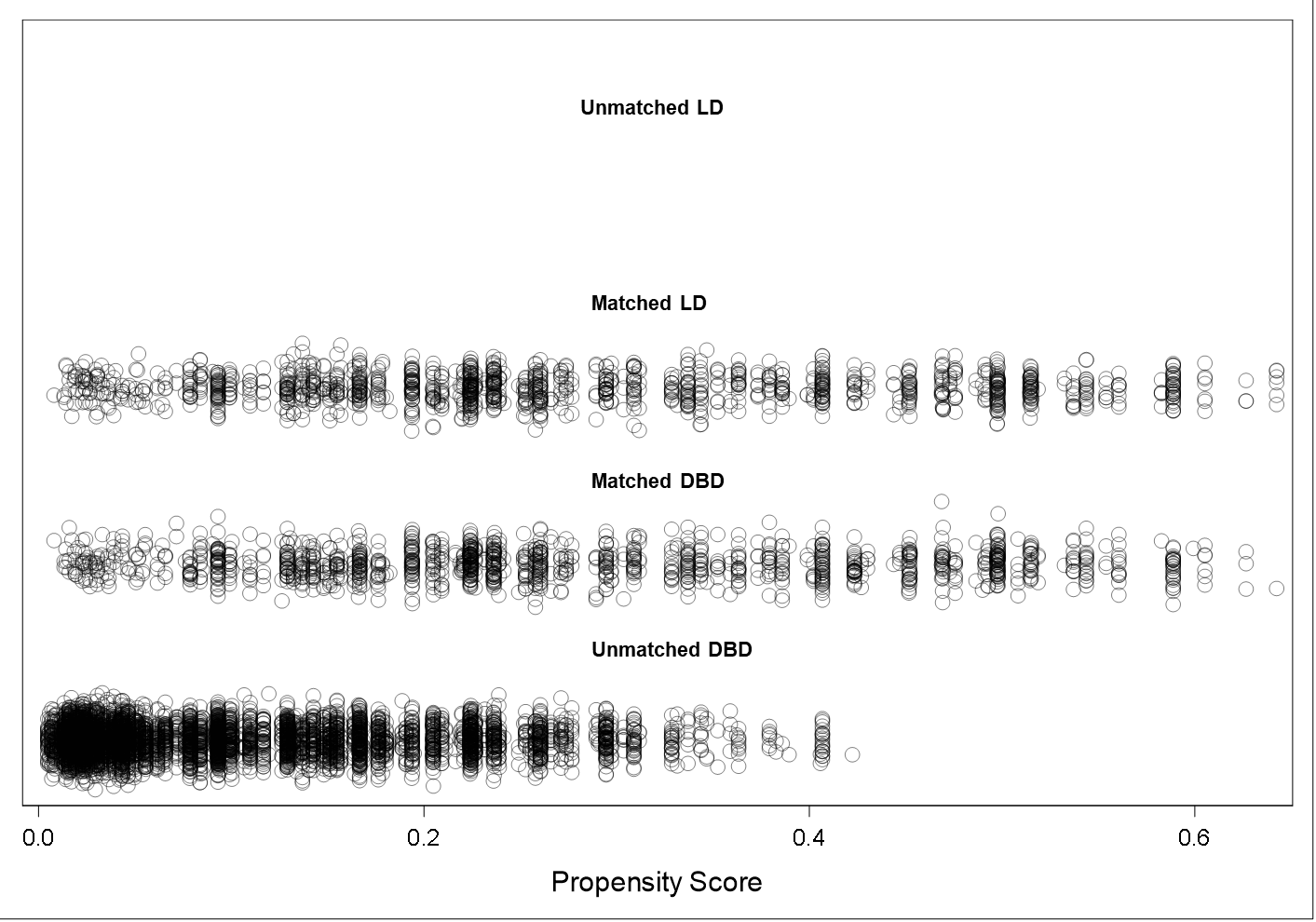


Note: No LD recipient was unmatched

LD: living donor,DBD; donation after brain death

**Supplementary Table S3. Univariate analysis of recipient high eGFR at 1 year (*) in the matched cohort (generalized estimating equation model stratified by pairs)**

|  |  | **N** | **n** | **% of events** | **OR** | **95% CI** | **p-value** |
| --- | --- | --- | --- | --- | --- | --- | --- |
| **Type of donor** | DBD | 2780 | 7506 | 25.81 | 1 | - | <0.0001 |
|  | LD |  | 1390 | 41.94 | 1.36 | 1.18–1.57 |  |
| **Recipient age** | 18-39 years | 2780 | 1714 | 60.33 | 6.83 | 5.38–8.66 | <0.0001 |
|  | 40-54 years |  | 2660 | 33.61 | 2.56 | 2.02–3.25 |  |
|  | 55-59 years |  | 1100 | 21.91 | 1.92 | 1.42–2.60 |  |
|  | ≥ 60 years |  | 3422 | 10.26 | 1 | - |  |
| **Recipient sex** | Male | 2780 | 5696 | 29.25 | 0.97 | 0.82–1.15 | 0.73 |
|  | Female |  | 3200 | 26.69 | 1 | - |  |
| **Recipient blood group** | A | 2780 | 3927 | 27.17 | 1 | - | 0.044 |
|  | AB |  | 405 | 32.1 | 1.81 | 1.22–2.68 |  |
|  | B |  | 965 | 31.19 | 1.06 | 0.82–1.37 |  |
|  | O |  | 3599 | 28.4 | 1.01 | 0.84–1.20 |  |
| **Recipient body mass index** | Underweight (<18.5 kg/m2) | 2780 | 358 | 51.4 | 2.27 | 1.58–3.26 | <0.0001 |
|  | Normal (18.5-24 kg/m2) |  | 3840 | 34.4 | 1.74 | 1.48–2.05 |  |
|  | Overweight (>= 25 kg/m2) |  | 4698 | 21.6 | 1 | - |  |
| **Duration of dialysis before transplantation** | Preemptive transplantation | 2780 | 1466 | 33.97 | 0.99 | 0.84–1.17 | 0.68 |
|  | <3 years |  | 4354 | 29.49 | 1 | - |  |
|  | > 3 years |  | 3076 | 23.99 | 0.87 | 0.64–1.19 |  |
| **cPRA** | 0% | 2780 | 5242 | 28.02 | 1 | - | 0.83 |
|  | 1-84% |  | 3016 | 29.94 | 1 | 0.85–1.19 |  |
|  | 85-100% |  | 638 | 23.2 | 0.87 | 0.53–1.40 |  |
| **Cause of ESRD** | Chronic glomerulonephritis | 2780 | 1936 | 31.77 | 1 | - | <0.0001 |
|  | Diabetes (type I or II) |  | 913 | 19.17 | 0.66 | 0.47–0.94 |  |
|  | Kidney malformation or hereditary nephropathy |  | 405 | 43.95 | 1.33 | 0.95–1.87 |  |
|  | Chronic interstitial nephropathy |  | 887 | 27.73 | 0.84 | 0.64–1.10 |  |
|  | Nephroangiosclerosis |  | 904 | 19.91 | 0.46 | 0.33–0.65 |  |
|  | PKD |  | 1501 | 25.38 | 0.78 | 0.62–0.99 |  |
|  | Others |  | 2346 | 31.67 | 0.94 | 0.76–1.16 |  |
| **Recipient cardiovascular comorbidities** | No | 2780 | 7602 | 30.04 | 1 | - | 0.0007 |
|  | Yes |  | 1294 | 18.24 | 0.64 | 0.49–0.84 |  |
| **Recipient diabetes** | No | 2780 | 7235 | 30.42 | 1 | - | 0.001 |
|  | Yes |  | 1661 | 19.21 | 0.68 | 0.54–0.87 |  |

(*) High eGFR at 1 year is defined by eGFR ≥ 60 ml/min/1.73m²

OR: odds ratio; CI: confidence interval; ESRD: end-stage renal disease; PKD: polycystic kidney disease; cPRA: calculated panel-reactive antibodies; eGFR: glomerular filtration rate estimated with the CKD-EPI formula ml/min/1.73m²; LD: living donor; DBD: donation after brain death

Supplementary Figure S2. Box plot of donor age according to recipient age and type of donation in the matched cohort


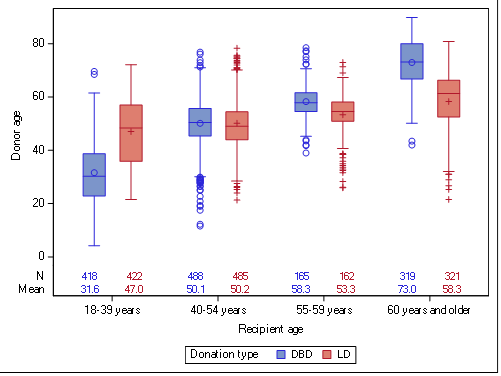


Supplementary Figure S3. Distribution of recipient eGFR at 1 year in the matched cohort by type of donation


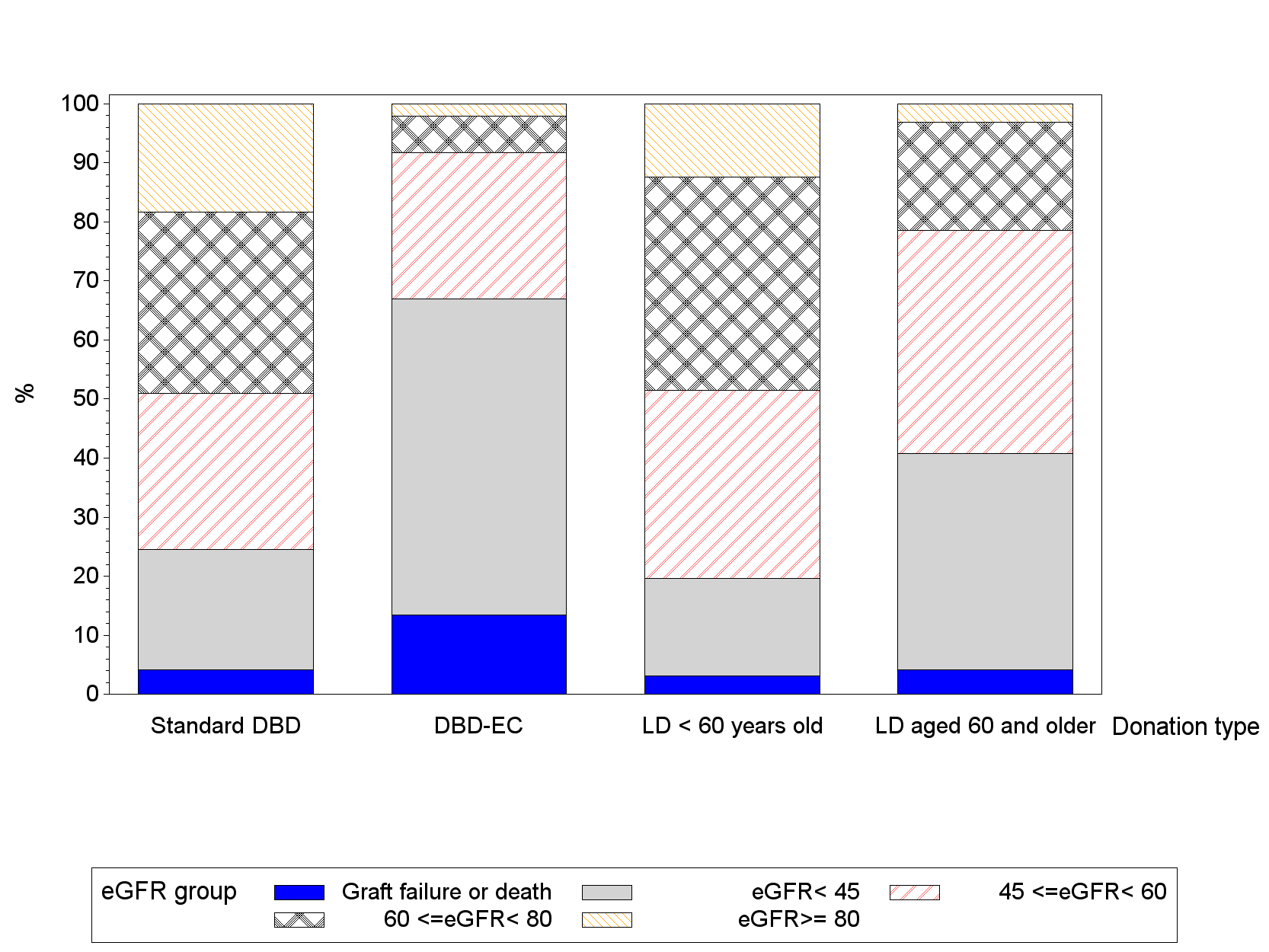


Supplementary Table S4. Description of continuous variables studied

|  | **DBD** | | | | **LD** | | | | **pvalue*** |
| --- | --- | --- | --- | --- | --- | --- | --- | --- | --- |
|  | **Mean** | **Median** | **1st interquartile** | **3rd interquartile** | **Mean** | **Median** | **1st interquartile** | **3rd interquartile** |  |
| **Whole sample** |  |  |  |  |  |  |  |  |  |
| Donor age | 57 | 59 | 47 | 70 | 51 | 52 | 44 | 60 | <0.0001 |
| Donor eGFR (c) | 73 | 74 | 54 | 93 | 95 | 95 | 87 | 104 | <0.0001 |
| Cold ischemia time (hours) | 16 | 15 | 12 | 19 | 4 | 2 | 1 | 4 | <0.0001 |
| HLA A-B mismatches | 2 | 2 | 1 | 2 | 1 | 1 | 1 | 2 | <0.0001 |
| HLA DR-DQ mismatches | 1 | 1 | 1 | 1 | 1 | 1 | 1 | 1 | 0.005 |
| Delta donor age - recipient age | 2 | 2 | -2 | 7 | 3 | 0 | -5 | 11 | 0.02 |
| Recipient age at graft | 55 | 57 | 45 | 66 | 48 | 49 | 37 | 59 | <0.0001 |
| Recipient BMI at graft | 26 | 25 | 22 | 29 | 25 | 25 | 22 | 28 | <0.0001 |
| Duration of dialysis | 3 | 2 | 1 | 4 | 1 | 0 | 0 | 1 | <0.0001 |
| Recipient eGFR at 1 year (mL/min/1.73 m2) | 50 | 47 | 34 | 62 | 58 | 57 | 46 | 68 | <0.0001 |
| **Matched sample** |  |  |  |  |  |  |  |  |  |
| Donor age for recipients aged 18–39 years | 32 | 30 | 23 | 39 | 47 | 48 | 36 | 57 | <0.0001 |
| Donor age for recipients 60 years and more | 73 | 73 | 67 | 80 | 58 | 61 | 53 | 66 | <0.0001 |

** pvalue from Student test*

Supplementary text S2. Final sensitivity analysis method

A final sensitivity analysis was conducted by considering all our continuous variables without categorizing them.

Therefore, we:

- constructed a second propensity score (PS2) by studying type of donor according to according to all our *a priori* variables: age, BMI, duration of dialysis as continuous variables, sex, blood type, cardiovascular morbidities, diabetes, calculated panel-reactive antibodies (cPRA). We used restricted cubic splines for our continuous variables following Harrell's recommendations by "placing knots at fixed quantiles (percentiles) of a predictor's marginal distribution" (page 26 Regression modeling strategies, Harrell):
  - recipient age: 4 knots (quantiles 0.05 0.35 0.65 0.95)
  - duration of dialysis, BMI: 3 knots (quantiles 0.10 0.50 0.90)

We kept cPRA as a categorical variable because of its strong interaction on the renal transplant allocation system and we also included all the candidate variables in our model.

Matching using this propensity score will hereafter be called Match_PS2.

- considered the eGFR as a continuous outcome, imputing an eGFR of 15 (threshold for stage 5 renal failure) for the 110 missing eGFRs of patients with graft failures (3.9% of the matched sample) and the 36 deceased recipients with a missing eGFR (1.3% of the matched sample).
- built a new model to test the consistency of the results, called new_model, we presented:

continuous eGFR at 1-year according to all our *a priori* variables: age, BMI, duration of dialysis as continuous variables with restricted cubic splines (constructed as for PS2), initial disease, cPRA in classes, blood group, sex, cardiovascular complications and an interaction between age and type of donor on the cohort matched Match_PS2.

*Note: we also considered continuous eGFR, imputing an eGFR of 61 to the 36 recipients who died with a missing eGFR, but as the results were the same than those with the imputation of 15, we do not present them.*

Supplementary Figure S4. Prediction from new_model of eGFR (mL/min/1.73 m2) at 1 year according to recipient age at graft in the matched cohort Match_PS2


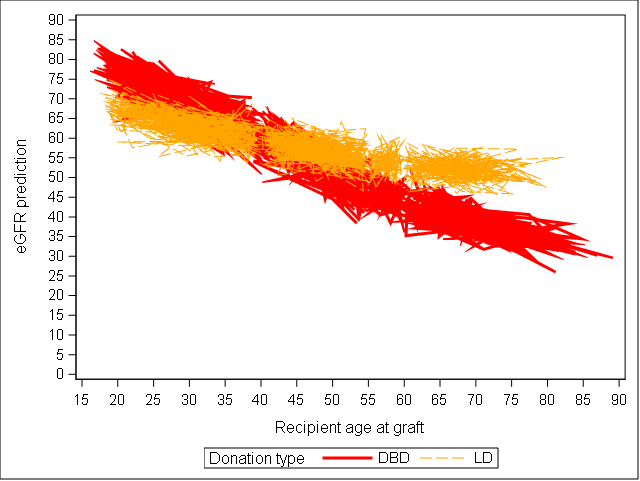


Capsule Sentence Summary

Older recipients benefit more from living donor kidney transplants compared to younger recipients, as demonstrated in an emulated trial involving living and brain-dead donor.
